# Beyond ideals: why the (medical) AI industry needs to motivate behavioural change in line with fairness and transparency values, and how it can do it

**DOI:** 10.1007/s00146-023-01684-3

**Published:** 2023-05-20

**Authors:** Alice Liefgreen, Netta Weinstein, Sandra Wachter, Brent Mittelstadt

**Affiliations:** 1 Hillary Rodham Clinton School of Law, University of Swansea, Swansea, SA2 8PP UK; 2 School of Psychology and Clinical Language Sciences, University of Reading, Whiteknights Road, Reading, RG6 6AL UK; 3 Oxford Internet Institute, University of Oxford, 1 St. Giles, Oxford, OX1 3JS UK

**Keywords:** Artificial intelligence, Healthcare, Medicine, Fairness, Bias, Motivation, Behaviour change

## Abstract

Artificial intelligence (AI) is increasingly relied upon by clinicians for making diagnostic and treatment decisions, playing an important role in imaging, diagnosis, risk analysis, lifestyle monitoring, and health information management. While research has identified biases in healthcare AI systems and proposed technical solutions to address these, we argue that effective solutions require human engagement. Furthermore, there is a lack of research on how to motivate the adoption of these solutions and promote investment in designing AI systems that align with values such as transparency and fairness from the outset. Drawing on insights from psychological theories, we assert the need to understand the values that underlie decisions made by individuals involved in creating and deploying AI systems. We describe how this understanding can be leveraged to increase engagement with de-biasing and fairness-enhancing practices within the AI healthcare industry, ultimately leading to sustained behavioral change via autonomy-supportive communication strategies rooted in motivational and social psychology theories. In developing these pathways to engagement, we consider the norms and needs that govern the AI healthcare domain, and we evaluate incentives for maintaining the status quo against economic, legal, and social incentives for behavior change in line with transparency and fairness values.

## Introduction

Forms of artificial intelligence (AI) have been adopted across the healthcare sector to augment the accuracy, efficiency, and quality of information feeding into healthcare decision-making (Haleem et al. 2019). AI is increasingly a trusted resource used by clinicians to make diagnostic and treatment decisions—assisting in imaging, diagnosis, risk analysis, lifestyle monitoring and health information management (Davenport and Kalakota 2019). This paper explores conclusions drawn within the AI healthcare literature that, given the trust healthcare practitioners place in AI, extensive investment is required to align AI technologies with certain values and ethical principles, to ensure they meet their end goal of improving medical care. These ethical principles resemble classical principles of bioethics and comprise, amongst others, justice (encompassing fairness) and transparency (Gabriel 2020; Royakkers et al. 2018).^1^

Despite their recognition, there is a lack of research investigating how to practically implement and integrate these principles within the AI development cycle—a process that requires trade-offs and overcoming a multitude of challenges (Ayling and Chapman 2021; Morley et al. 2021; Vakkuri et al. 2019; Whittlestone et al. 2019). While value alignment^2^ has gained increasing attention, it is primarily approached from a normative and technical standpoint (Gabriel 2020; Stray et al. 2021; Sutrop 2020), and has largely neglected the need to foster alignment among the values and ethical principles of the developers, stakeholders, and agents involved in the creation of the AI system with shared values, such as transparency and fairness. In this paper, we highlight the importance of developing strategies to motivate individuals and organizations to instil these values and ethical principles into their AI systems from the outset (Mittelstadt 2019; Morley et al. 2020; Weinstein et al. 2013; Whittlestone et al. 2019) and argue that this is a crucial step to creating long-lasting behavioral change that results in new bias mitigation technologies being used, not just made available, across the healthcare sector. Identifying weaknesses of AI systems and agreeing on principles these systems should reflect and abide by is not sufficient to ensure the technology used in healthcare domains (and beyond) is trustworthy or ethical (Mittelstadt 2019). Addressing these challenges necessitates extensive human investment in solving the problem of bias in technology. Critically questioning the effectiveness of current and future technologies to understand their limitations owing to bias and taking layered measures to change the way that technology is designed and implemented requires sustained human effort and the willingness to risk significant reputational, project management, and economic costs. In addition, we argue that the effort required to truly understand and correct for bias in AI will be afforded only when driven by people’s ethics and values—their deeply-held worldviews regarding what is the important and right thing to do.

To identify means of addressing these challenges, we conducted a review of the literature on topics including the identification and mitigation of biases in AI systems, behavior change interventions, value systems theory, self-determination theory and AI and ethics. We brought together multiple perspectives from specialists in the fields of AI law and ethics, data ethics and philosophy, and behavioral and motivational psychology. We then synthesized these perspectives in the context of existing literature and frameworks from these interrelated fields. This enabled us to develop a thorough and nuanced understanding of the challenges and opportunities associated with translating ethical principles of fairness and transparency into practice and leverage this understanding to present possible solutions to these challenges.

This paper is structured as follows. We begin by examining some of the most common biases that permeate AI systems, such as data-driven bias, and discuss some of the solutions that have been implemented to address them, including data curation processes and auditing practices. Next, we turn our attention to means of promoting fair AI through increased transparency in the design and auditing processes of AI development and deployment—arguing that this requires a deeper understanding and leveraging of the values that drive decisions in AI development. Subsequently, we delve into the field of motivational psychology—which focuses on understanding how social environments influence behavior and developing strategies for promoting behavioral change (e.g., Deci and Ryan 2008)—to promote trustworthy AI technology. More specifically, we examine the potential of self-determination theory, autonomy-supportive strategies, value salience with identity, and the approach-avoidance framework to inspire behavior change and bridge the gap between values and behavior regarding trustworthy AI. Finally, we place these strategies in context by considering the enablers and barriers to behavior change, such as the role of social norms and the economic advantages of maintaining the status quo, as well as the financial and regulatory incentives of developing effective AI governance solutions to promote fairness in AI technology.

By bringing together insights from the fields of psychology, AI, philosophy, and computer science, we hope to provide a holistic understanding of the challenges and potential solutions for promoting trustworthy AI in healthcare and beyond.

## Bias in healthcare technology

Benefits of healthcare technology are wide-ranging and far-reaching—including increased speed and accuracy in imaging, diagnostics for patients suffering from complex diseases, more accurate predictive screening and prognosis, and overall increased efficiency (Davenport and Kalakota 2019; Esmaeilzadeh 2020; Yoon and Lee 2021). For example, IBM developed the *Watson Care Manager System* to improve cost efficiency, design individually tailored healthcare plans, and aid in the effective use of managerial resources (Sun and Medaglia 2019). Elsewhere in clinical care, the *Ultranomics system* uses AI to analyze echocardiography scans that help detect patterns in heartbeats and diagnose coronary heart disease (Dinakaran and Anitha 2018). AI technology has also been adopted within radiology, with the development of systems able to automatically detect and quantify, as well assess, the growth and classification of abnormalities in CT scans. These systems promise greater accuracy and efficiency in prediction and prognosis compared to human radiologists using the naked eye (Jalal et al. 2021).

Despite these benefits, similarly to other domains in which AI has been in use, bias and discrimination have been flagged as key risks facing AI applications in healthcare.^3^ Groups which have historically faced healthcare inequalities, including minority racial and ethnic groups, women, lower-income patients, and other underrepresented or disadvantaged groups, face similar challenges with medical AI tools (Chen et al. 2022; Wachter et al. 2021b). Within this domain, biases can result from long-standing societal prejudices and inequalities amplified by problematic datasets and AI models that learn from them (Parikh et al. 2019a, b; Wachter et al. 2021a, b). There are a wide range of instances in which algorithms make spurious associations between protected factors, such as race, and disease outcome, when the underlying causal factor stems from social determinants of health (e.g., lack of access to care, delayed screening, insurance type) and not the class of a protected factor itself (Chen et al. 2022; Obermeyer et al. 2019; Vyas et al. 2020). The medical field has numerous examples where racial, gender or age disparities affect clinical decision-making, quality of treatment, and diagnosis (Norori et al. 2021; Webster et al. 2022). For example, it is recognized that Black patients have lower survival rates than white patients for different cancer categories, as well as cardiovascular disease (Lam et al. 2018; Norori et al. 2021; Tajeu et al. 2020). Nevertheless, AI systems used to predict the risk of cardiovascular disease are trained on datasets with a majority of (white) male cases, thereby displaying less accuracy for female patient groups or people of color (Antoniades and Oikonomou 2021; Norori et al. 2021).

Another example of data-driven bias in healthcare involves polygenic risk scores that use data from genome-wide association (GWAS) studies to calculate a person’s inherited proneness to disease (Norori et al. 2021). Although a polygenic risk score has excellent potential as a predictive biomarker, 81% of GWAS studies have been conducted in individuals of European ancestry (Popejoy and Fullerton 2016), affecting the score’s generalizability across different populations, and ultimately resulting in biased predictions that perpetuate inequalities in health outcomes. Relatedly, Larrazabal et al. (2020) recently provided empirical evidence supported by a large-scale study of a consistent decrease in algorithm performance for underrepresented genders when diagnosing various thoracic diseases under different gender imbalance conditions. When the software was reprogrammed with more gender-balanced data, it performed better by detecting thoracic diseases more accurately on X-rays, proving that more diverse datasets can improve the software’s clinical performance in a diverse patient population.

### Technical approaches to mitigating biases

Effective governance of AI systems, which ensures the delivery of equitable healthcare and mitigates the presence of bias, requires extensive effort on the part of those involved in the development and deployment of AI systems. Part of these efforts should be focused on tackling unfairness and discrimination which stems from training algorithms on skewed datasets, or datasets reflecting socio-economic and historical inequalities in our world.

One approach to mitigate these effects is to find ways to train models using datasets representing larger and more diverse patient populations (Food and Drugs Administration 2019). Training datasets should ideally be *diverse* along three dimensions: (i) *individual*, considering different biological factors, such as age, sex, and race; (ii) *population*, reflecting diverse disease prevalence, access to healthcare, and cultural factors; and (iii) *technical*, containing data originating from different types of medical machinery, using various acquisition or reconstruction parameters (Barclay 2021). A dataset should not only be diverse but also *balanced*, meaning it has an even distribution of a set of relevant features across the dataset. Despite the need for fair representation, clinicians often rely on publicly available datasets which are not representative of different sub-groups (Norori et al. 2021). This is because historically, vulnerable groups have been omitted from, or misrepresented in, medical datasets –meaning that AI systems trained on historical data have limited predictive ability when it comes to these groups (Parikh et al. 2019a, b). Obtaining raw medical data that is diverse and balanced is a major challenge for various reasons, including data protection regulations which tend to be particularly restrictive for health-related data (for discussion see Murdoch 2021).

Data curation and enrichment is another area which needs extensive human investment to ensure it does not contribute to training biased algorithms. AI typically needs large amounts of data to train and prepare for real-world applications. Before use, training datasets requires human curation and enrichment processes including filtering, cleaning, and labelling (Gerke et al. 2020). These processes can be very time-consuming but are essential to ensure the dataset accurately reflects a “ground truth” and is thus a valid foundation to train the model. In the healthcare domain, this can mean validating the output as a true positive or negative when the algorithm is in the learning phase (e.g., accurately labelling a tumor on a given X-ray). This labelling phase is carried out by experts within the domain and remains subjective and open to interpretation. For example, in radiology, where experts label scans used to train AI algorithms, individual differences arise in labelling practices. Experienced pathologists will often disagree about histopathology and diagnosis, particularly early-stage lesions. This level of human bias, which introduces subjectivity to the dataset’s “ground truth,” can lead to biased models and outcomes (Willemink et al. 2020).

### Auditing practices to mitigating biases

AI technology is prone to bias and yet is trusted by medical practitioners to assist their practice (Juravle et al. 2020). Recognizing this problem, AI auditing frameworks have been developed to advocate for transparency in recounting an AI system’s development and deployment. This includes reporting all collected artefacts, datasets used, test results, risk mitigation plans, and final decisions made, as well as any changes made to the AI system following an audit (Liu et al. 2022; Oala et al. 2021). Recognizing bias and the need for greater awareness, *toolkits* based on these frameworks have been put forth to evaluate the fairness or bias of machine learning models and automated decision-making/prediction systems. These toolkits monitor bias and unfairness issues throughout a model’s lifecycle (from early production to implementation), thus facilitating the appraisal of an algorithm’s performance as well as its failings (Morley et al. 2021). Ultimately, they are designed to help developers, policymakers, and laypeople obtain a greater understanding of the limitations and functionality of AI systems and move toward the development of fairer algorithms that do not perpetuate discriminatory behavior (Stevens et al. 2018, 2020; Zehlike et al. 2017).

Recently, auditing frameworks tailored to medical AI systems have also been advanced. These aim to guide the auditor (i.e., a developer or user, such as a medical practitioner) through a systematic process of considering potential algorithmic errors—defined by Liu et al. (2022) as any outputs of the AI system which are inaccurate, including those inconsistent with the expected performance of the system—in the context of a given clinical task, mapping the factors that might contribute to the occurrence of errors, and anticipating their potential consequences (Liu et al. 2022; Oala et al. 2021; Panigutti et al. 2021). These frameworks additionally outline approaches for testing algorithmic errors via, for example, sub-group testing or exploratory error analyses (Liu et al. 2022). The aim is to take into consideration a dynamic set of technical, clinical, and regulatory considerations found in law and outlined in regulatory bodies when evaluating medical AI systems. This approach highlights the need to go beyond providing solely quantitative performance measures to tackle issues of bias, and also offer qualitative ones (Oala et al. 2021). This comprehensive practice can help auditors and users identify ways to mitigate the impact of weaknesses in their technology at various levels, such as modifying the artificial intelligence model, modifying the model threshold, or modifying the instructions for its use (Liu et al. 2022; Oala et al. 2021).

#### Transparent communications in auditing practices

Transparency is a key value that, if activated in developers of the technology, can drive increased engagement in creating fair systems. So far, there are mixed findings on the effect of reporting measures of uncertainty, or limitations in performance, directly to users of AI systems. The majority of this research, however, has focused on the influence of this information on healthcare practitioners’ levels of *trust*—finding in some cases that information on a prediction’s uncertainty fosters trust in users, and in others reporting it hinders the development of trust (Cai et al. 2019a; b; Glass et al. 2008; Ha et al. 2020; Kim and Song 2022; Papenmeier et al. 2019; Robinette et al. 2017; Vorm 2018).

However, the possible benefits of transparently communicating auditing results were recently empirically demonstrated in a study carried out by Raji and Buolamwini (2019). The authors selected target companies from the original Gender Shades Study^4^ (Buolamwini and Gebru 2018), as well as companies not targeted within the initial audit, and conducted a follow-up audit on these companies to test whether there had been improvement in model performance across identified subgroups which fared poorly in the original study. The findings of this follow-up audit revealed universal improvement across intersectional subgroups in the systems utilized by all targeted companies. Although post-audit performance for the minority class (darker-skinned females) was still the worst relative to other classes, the gap between this subgroup and the best performing subgroup (lighter-skinned males) reduced significantly after companies released updates following the initial audit (Raji and Buolamwini 2019). Additionally, the authors found that minimizing subgroup performance disparities did not jeopardize overall model performance but rather improved it, highlighting the alignment of fairness objectives to the commercial incentive of improved performance and accuracy (Raji and Buolamwini 2019).

While further research is needed on this matter, knowledge of risks and a system’s performance is arguably an important prerequisite to achieving the goals of transparency, increasing autonomy and control for users of AI systems, and facilitating accountability practices. Research should also focus on identifying the factors that drive organizations to address algorithmic bias proactively and engage in auditing practices in the first place. This will facilitate the development of frameworks to improve engagement and awareness, improve the efficacy of algorithmic auditing practices, and formalize procedures for the transparent communication of a system’s development, performance, and evaluation.

## How to promote investment in fair and transparent technology

Advocates of technical solutions (including toolkits) to obtain fair and trustworthy AI technology, have increasingly recognized that only through disclosure and transparency in the design and auditing process will AI solutions improve over time. Yet, there is a notable gap in our knowledge base. Whereas plenty of attention has been placed on identifying where biases lie within AI systems, and on developing technical solutions to mitigate their presence, comparatively less attention has been placed on evaluating the effectiveness of these existing approaches and developing strategies to incentivize individuals and organizations to instill fairness into their AI systems and development processes from the outset (Burr et al. 2020; Crawford 2016; Jobin et al. 2019).

Companies developing and using AI have put forth a variety of information campaigns. These primarily communicate the presence of bias in AI. They also describe where issues lie, and which aspects of the technology development and implementation process are most vulnerable to bias. Some benefits of this approach can be recognized in the positive effect of public audits, partly attributed to increased corporate and public awareness of the problematic discriminatory consequences of the algorithms which incited the companies to speedily release product updates (Raji and Buolamwini 2019). In addition, literature in computer science has previously cited this notion of promoting fairness through user awareness and education (e.g., Hamilton et al. 2014). The approach of recognizing the dangers of AI systems and communicating these to relevant stakeholders undoubtedly has merit. However, research demonstrating that raising awareness leads to behavior change in this domain remains scarce.

Although important, knowledge of an issue alone is often not enough to lead to behavior change (Arlinghaus and Johnston 2018). This is a perception that is not new in the field of psychology, with much research showing that motivating behavior change requires more than providing knowledge and awareness of the behavior that needs to be changed (Arlinghaus and Johnston 2018; Corace and Garber 2014; Feldman and Sills 2013; Hussein et al. 2021). Rather, prior research has shown that to ensure sustained behavior change, one needs to engage with the target subjects in ways that elicit their own goals, interests, and plans so that they can develop their value for, and interest in, the positive behavior (Connell et al. 2019; Kullgren et al. 2016; Patrick and Williams 2012; Teixeira et al. 2011).

As a result of the wave of research identifying issues relating to bias within AI systems and identifying the legal and ethical principles which AI systems should follow, there is now widespread agreement around the basic principles that ethical and fair AI should meet in healthcare domains and beyond. These include beneficence, non-maleficence, autonomy fairness, explainability, accountability, and understandability (see Jobin et al. 2019; Royakkers et al. 2018). To promote behavior change in line with creating AI systems that abide by these principles and are representative of human values, we argue for a need to understand, and subsequently leverage, the values that underlie decisions made by individuals involved in crafting and deploying these systems.

Developers play a vital role in the pipeline of an AI system, as they are involved in data pre-processing, parameter and model selection, and are responsible for making model architecture changes to fit a particular use. Communicating to developers about the different biases that creep into the AI pipeline is useful to raise their awareness of these issues and enable them to address fairness limitations. However, the effectiveness of these communications in translating to behavioral change will depend on their content and on contextual and individual factors relating to, for example, values and beliefs. Understanding these factors and leveraging them when issuing communications about bias in AI and developing de-biasing strategies could be an essential means to effectively translate increased awareness of the issue into behavior change. This entails viewing investing in fair AI as a value-expressive behavior, building on previous work that sees certain behaviors as giving expression to particular values (Bardi and Schwartz 2003).

### Behavior change and values

Values are stable characteristics that describe what people hold most *important* and guide attitudes and behaviors, the latter of which are responsive to contexts and changing situations (Feather 1990; Maio and Olson 1994; Rokeach 1973). Even among large commercial organizations, individuals’ values drive decision-making that ultimately produces value-congruent or value-incongruent products. In the context of healthcare technology, developers are the ones to instantiate values in AI systems via, for example, dataset curation and enrichment (Gerdes 2022; Sanderson et al. 2022). Relatedly, the values of an organization influence how discrimination is defined within the organization and what solutions are developed to tackle its presence. Therefore, understanding the values and norms of developers and members of AI organizations is key to promoting fairness by design—the practice of designing and deploying AI solutions that reflect and promote the values and ethical principles shared by society from the outset. This approach moves away from adopting reactive approaches to address issues of fairness and bias after an AI system is completed, and closer to a proactive method oriented toward infusing the systems with these shared values from the outset.

Given that technologies inevitably embody values (Nissenbaum 2001), pre-emptive attention to values during the design stage can ensure that the process and the final product (e.g., AI system) reflect these values in the long run (Felzmann et al. 2020). This is a key principle behind ethical AI design, including approaches like safe-by-design (SBD) (Baum 2016), value-sensitive design (VSD) (Friedman et al. 2013; Umbrello 2019; Umbrello and van de Poel 2021; van den Hoven et al. 2015a, b), and value alignment solutions (Gabriel and Ghazavi 2021a, b; Yudkowsky 2011)—methodologies that hold the premise that technologies are value-laden and that human values are implemented during and after their design (Friedman et al. 2013).

Social and moral psychology theories such as value systems theory, also stress the importance of focusing on the values and principles of those involved in creating and deploying AI systems. By linking ethical principles to personal and organizational values through values theory, organizations can inspire their employees to act in accordance with these principles, leading to a culture of ethics and accountability that supports the consistent implementation of transparency and fairness in AI systems. Literature in organizational psychology has evidenced that the values and motivations of individuals are crucial to reducing bias and discrimination within organizations—and that corporate efforts at reducing discrimination are ineffective without motivated individuals (Dobbin and Kalev 2018; Sullivan et al. 2001). Similarly, individuals are more likely to adopt changes in policy or practice if they perceive an organizational shift in line with their values (e.g., see Angehrn 2005). These notions remain essential when considering ways of eradicating discrimination from AI systems and creating and rolling out solutions that promote fairer and less biased AI systems.

A practical dimension of values from psychology contrasts between self-transcendent values, those that reflect care and concern for others and the world outside of oneself, and self-enhancing values, those that represent a focus on oneself, for example, in terms of increasing one’s success or sense of power (Schwartz 1992, 2012). Taxonomies of specific values distinguished in terms of where they lie on this model are highly consistent across many cultures (Schwartz 1992). The distinction between the two categories of values—self-transcendent values that comprise universalism (caring for others equally) and self-enhancing—helps to predict whether value-congruent behavior will be undertaken (Nordlund and Garvill 2002; Schoenefeld and McCauley 2016).

Most who have been queried in cross-cultural samples endorse the self-transcendent values—universalism (caring for others equally) and benevolence (value of enhancing the welfare of the community; Schwartz 2012)—needed to invest in trustworthy and fair technology (Bardi and Schwartz 2003). In principle, it should be easy to get people to do the ‘right’ thing regarding trustworthy technology. So, why are there still such prominent and unacknowledged biases in technology? Studies show a discrepancy between people’s stated values and their behaviors, and small to moderate relations have been reported linking values and corresponding behaviors (Bardi and Schwartz 2003; Cieciuch 2017; Schwartz et al. 2017; Schwartz and Butenko 2014). This value-behavior gap is an elusive foe. Despite efforts to identify sources for eliminating it taken over many years, it has historically escaped researchers’ actions in several value-driven behavior domains, such as in environmental behaviors (Kollmuss and Agyeman 2002; Linder et al. 2022), prosocial behavior (Dovidio et al. 2017) and prejudice reduction (Paluck et al. 2021). The difficulty arises in part because values exist in the abstract. In contrast, specific situations have concrete needs and challenges that may prevent them from being consciously associated with their corresponding values or that may create barriers to change. Put differently, whereas an ideal situation would be to follow one’s values consistently, one may fail to do so because they fail to recognize how their values could best be expressed in a specific situation or because they feel blocked in expressing their values because of the current demands of the situation (Maio 2010).

Further, to change one’s existing behavior patterns to align these with values, one must change habits (Legate et al. 2021; Legate and Weinstein 2021, 2022). The ways that people apply their values to their day-to-day work and life become habitual through repetition. Certain decisions and priorities are familiar through their recurrence in the work environment; certain ways of talking about and evaluating priorities become normative. Those behaviors that may not be well-aligned to one’s values have been reinforced in conditions where contradictory outcomes are appreciated at the institutional level (Legate and Weinstein 2022). Therefore, these habits must be re-examined, and new information integrated to change the output. To do so, we must consider the wider professional environment in which people operate, and the barriers it might raise to engaging in value-consistent behavior.

It is now recognized that strategies for changing behavior must be sensitive to the context of the behavior (for example, recognizing existing workplace cultures or practical challenges of undertaking behavior; Hutchison 2019; Miner and Costa 2018; Pless and Maak 2004), consider employing multiple strategies at once to produce change (Coleman and Pasternak 2012), and ultimately test sustained behavioral change (Volpp and Loewenstein 2020). A recent study by Winecoff and Watkins (2022) emphasized that the discordant values of start-up entrepreneurs (and developers) and their funders often hindered the product’s (AI system) ability to reflect their values. Findings emphasized that while entrepreneurs aimed to preserve their values—especially those connected to scientific integrity and organizational autonomy within their organization—the demands of technology entrepreneurship often ran counter to these values by focusing predominantly on rapid innovation and financial gain. In this case, the need to operate within the fast-moving technology industry constrained how entrepreneurs and developers fully transformed their ethical values into substantive practices. Whittlestone et al. (2019) recently argued for the need to focus research attention not only on identifying principles and values that AI should follow, but also on identifying the tensions that inevitably arise when implementing these principles in practice—such as tensions between the goals of an AI system and the risks it introduces to other values, or the tensions between the interests and values of an organization, a user, and a developer. Understanding and leveraging the values of developers and the environment in which the AI system is being developed is an important step to developing effective solutions to the problem of biased AI.

## What motivational psychology can tell us about behavior change

Tackling these various social challenges, in motivational psychology, strategies have been devised that reduce the gap between values and behavior. Motivational approaches try to package or frame information in ways that increase the effectiveness of information being delivered to encourage sustained engagement. These can be applied to inspiring behavior change in line with fairness and transparency values in the service of trustworthy AI technology.

One approach to framing information to motivate change comes from self-determination theory (SDT; Deci and Ryan 1985). SDT posits that value-consistent behavior, such as, in our case, decisions to promote fairness or transparent communications, is undertaken when they are energized by different and distinct motives that reflect reasons which are autonomous and self-driven (i.e., internalized and influenced by one’s interests and values) or controlled and other-driven (i.e., external and influenced by expectations and pressures from the environment; Ryan and Deci 2017). Inspired by SDT theory, there has been significant interest in identifying autonomy-supportive strategies for conveying information. Much of this information has been in the context of healthcare (Altendorf et al. 2019; Legate et al. 2021; Moon and Woo 2021; Moon et al. 2021) sports, and education (Reeve 2016; Vansteenkiste et al. 2004a, b; Vansteenkiste et al. 2004a, b). These survey and experimental studies have shown that promoting internalized, autonomous motivation through autonomy-supportive strategies—providing a rationale to convey the importance of the behavior being motivated, creating a sense that one has a choice about how to behave, and avoiding using pressure and shame—are more effective at sustained behavioral change (see Legate and Weinstein 2021, 2022). This is because they give those being motivated the psychological space to consider their own (rather than the required) commitment to their values and whether their behaviors align with those values, while simultaneously inspiring personally meaningful reasons to build their commitment in the first place.

One of the ways of creating autonomous motivation for action is by pairing value salience with identity. Indeed, previous research shows that values are linked to behavior insofar as they are central to identity (Verplanken and Holland 2002). In the context of environmental behaviors, identity is seen as a core driver for environmentally friendly ‘green behaviors’, especially in cases where those behaviors come at a cost or challenge (Jaspal et al. 2014). In healthcare contexts, researchers have also proposed that small shifts in identity open the door to seeing the possibility of change, reinforcing this change, and subsequently supporting even more behavior change (Kearney and O’Sullivan 2003). Much like is the case for values, individuals who engage in certain behaviors can distance their behaviors from their identity. For example, someone can hold implicitly prejudiced beliefs but not self-identify as racist (Archakis et al. 2018). In healthcare, similar observations have been made regarding addiction—addictive behavior can be compartmentalized from people’s identities as addicts (e.g., Choi et al. 2010). From an SDT perspective, having an identity consistent with one’s values helps the behavior to be fully autonomous, in a form called integrated motivation. Behaviors motivated by integration reflect the core aspects of the self—they are aligned with the internal drivers that energize behaviors (Deci and Ryan 2008).

Other theories in the fields of social psychology support this point. For example, cognitive-dissonance theory suggests that people will try to reduce discomfort caused by holding conflicting beliefs (Festinger 1957). In the context of building AI systems, developers may have to reconcile their personal values with the ethical considerations of designing a given system, to minimise cognitive dissonance. Relatedly, the approach-avoidance framework outlines that individuals are motivated to approach stimuli or situations that are associated with positive outcomes and to avoid stimuli or situations that are associated with negative outcomes (Elliot and Thrash 2002). These areas of research suggest that an effective motivational strategy could involve highlighting the alignment of ethical principles of fairness and transparency with positive outcomes, such as increased trust in the AI system, enhanced reputation of the organization, and improved stakeholder satisfaction.

Recent literature evaluating the challenges of implementing ethical principles in practice (e.g., see Goirand et al. 2021 for a discussion relating to the healthcare industry and Mökander and Sheth, (2023) to the biopharmaceutical industry) has identified a range of difficulties, including a lack of clear governance structures, limited understanding of AI, ethical dilemmas, difficulty in aligning governance principles with business goals, balancing competing interests, legal and regulatory challenges, lack of standardization and best practices, and limited engagement with external stakeholders. Strategies based on motivational psychology theories, such as self-determination theory and value systems theory, might offer potential solutions to some of these challenges. Self-determination theory can enhance intrinsic motivation by aligning personal and organizational values, promoting a collaborative culture, and providing employees with a sense of control and autonomy. This can lead to improved ethical decision-making and more effective implementation of AI governance principles. Relatedly, other strategies stemming from motivational psychology principles, such as involving stakeholders in the design and implementation process, providing transparency and control over data use, and fostering a sense of community and collaboration—can help align AI-based applications with ethical principles such as transparency, privacy, and fairness.

## Barriers to behaviour change

It is useful to draw on these principles based on social psychology to develop effective AI governance solutions and motivate individuals to invest in them. However, these principles do not exist in a vacuum. To translate them into practice, we must consider the norms and needs that govern our context of interest, and which might act as barriers to change. This will enable us to operationalize value-activation interventions aimed at incentivizing the adoption of tools that promote fairness in AI technology.

### Norms

In the values literature, one of the notable drivers of inaction is that even when people hold self-transcendent values that should drive their positive behaviors, they perceive others to lack those same values, contributing to a perception of helplessness to produce change (Sanderson and Dawe 2019). Thus, social norms and perceived social norms are crucial elements to consider when developing behavior change interventions, especially if the goal is to achieve sustained behavior change on a large scale. Though more than one definition exists, broadly, social norms can be thought of as standards for behavior that people utilize to guide their behavior and evaluate that of others (Smith 2020). Individuals often have norms that define the kind of behavior they should or should not engage in. Similarly, organizations have norms that will dictate how members of the organization should behave and how the organization operates. Even more broadly, societies have shared beliefs about prescribed or banned behaviors.

These norms can be highly influential in guiding behavior at each level, via various channels. The *informational* influence of norms occurs when people conform to a behavior, such as wearing a mask, because actions of those in their social groups are indicative of these actions being the correct behavior. For example, they are convinced it is the right thing to do to reduce transmission risk of an infectious disease. *Normative* influence occurs when people conform to be accepted by their social group. For example, they wear a face mask to not stand out negatively (Neville et al. 2021). When discussing social norms, it is worth noting that these have both injunctive elements (defining what should be done) and descriptive elements (defining what is done). *Injunctive* norms refer to beliefs about what behavior is approved or disapproved of, and motivate behavior adoption using social rewards or punishments associated with the behavior. *Descriptive* norms describe the status-quo behavior and boost consensus to adopt it primarily by providing information about what behavior is likely to be effective and adaptive in a specific context (Neville et al. 2021).

Research on social norms and behavior change has shown that messages reinforcing positive norms can effectively change behavior, especially when evoking a shared identity (Reynolds et al. 2015). Descriptive normative communications centered around alerting users and organizations about the behavior of other similar individuals and organizations seem particularly effective in doing so in a range of contexts spanning from adopting pro-environmental behaviors (De Groot et al. 2013; Göckeritz et al. 2010; Goldstein et al. 2008; Nolan et al. 2008) to tax compliance behavior (Behavioural Insights Team 2012). In contrast to coercive approaches, manipulating norms in communications aims at producing bottom-up changes that will ultimately be adopted at a societal level (Bicchieri 2016). This approach seems valuable when considering solutions to promote fair AI and motivate behavioral change within the AI industry in favor of fairness by design.

### Economic advantages

The task of re-examining longstanding habits and changing one’s practice is challenging because normative decisions, even those resulting in biased technology, sometimes serve worthwhile functions—such as offering economic advantages. For example, Google’s combination of search and advertisement activities (e.g., advertising networks), has in the past decade come under scrutiny for resulting in conflicts of interest and incentives to bias search result rankings. Rieder and Sire (2014), argue that the company’s role as a search provider and a content provider strongly motivates it to organize search results in a biased and self-serving way. This, combined with its recognized dominance on the market, compounded in Google being repeatedly fined for abusing its dominance as a search engine and (i) imposing restrictive clauses in contracts with third-party websites which prevent rivals from placing their search adverts on these websites (European Commission 2019), and (ii) giving an unfair advantage to their comparison-shopping services (European Commission 2017).

Another example of when *not* addressing bias in AI systems can be an economic advantage relates to adverts for Science, Technology, Engineering and Mathematics (STEM) jobs. A research study found that women see fewer advertisements about entering STEM professions than men (Lambrecht and Tucker 2019).This is not due to companies purposefully targeting men in a disproportionate fashion, but rather because of economic ad sales. For advertisers, it is more expensive to get female views than male views on digital ads. This results in the creation of ad algorithms which, designed to save costs, end up targeting the cheaper male viewers (Lambrecht and Tucker 2019; Maron 2018). In this example, the primary issue seems to arise from having specific goals and objectives which are at odds with ensuring fair outcomes. The auction-based ad allocation system attempts to save advertisers money in their ad targeting; this might be a meaningful goal, but it places no value on fairness and gender equality.

Examples of this issue can also be found within the academic research domain. Big data obtained from social platforms such as Twitter have become increasingly popular for studying human behavior. This is not because it is a representative dataset (often this is not the case), but because it is the cheapest, fastest, and easiest to access for researchers. Researchers affected by time and funding constraints make use of Twitter as it is readily available and voluminous. In this case, questions about fairness and representativeness might take a backseat to other needs such as publication and funding.

### Financial costs of fairness tools

The financial costs of implementing fairness metrics in an algorithm or system are another critical inhibitor to consider when devising effective behavior change strategies. AI fairness metrics typically increase the prediction performance for the sensitive or protected group (Hardt et al. 2016; Zietlow et al. 2022)—this can mean lowering it for the non-protected group, which often represents the more extensive user base of a system (von Zahn et al. 2021). Hence, one could expect that implementing fairness metrics results in some financial costs. This has been recently empirically recorded in e-commerce (von Zahn et al. 2021). AI development still occurs largely within the commercial sector, which is guided by financial principles. Therefore, it is unsurprising that, in part, biased algorithms and biased datasets (e.g., unbalanced) are not a company’s primary concern unless perhaps they lead to financial deficits. Imagine that a company’s new vision AI system fails to recognize the imagery of certain cultural practices. If the communities that engage the most in these cultural practices are not its primary customer base, the company has little incentive to address those biases as doing so would not result in any economic benefits.

Similarly, implementing solutions such as auditing toolkits can incur both financial and administrative costs including investing time and resources into preparing for audits, as well as the costs of implementing any changes to the AI system, or its use, that come to light following the audit (Mökander and Floridi 2022). Recently, the European Commission estimated that certification for an AI system that abides by the EU AI Act could cost on average EUR 16,800–23,000, equating to approximately 10–14% of the development cost (Moritz et al. 2021; Norori et al. 2021). Others have argued this to be a conservative estimate (Haataja and Bryson 2021; Mueller 2021).

## Enablers of behavioral change

Nonetheless, there are also clear incentives to design and implement fair AI systems. These include improving various metrics such as data security, talent acquisition, reputational management, process optimization, economic advantage, and regulatory preparedness (Holweg et al. 2022; Mökander and Floridi 2022; The Economist Intelligence Unit 2020). Here, we consider two possible incentives that can be leveraged as enablers of behavioral change in line with designing AI systems imbued with transparency and fairness values: economic incentives and regulatory incentives.

### Economic(s) incentives

Adopting fair AI solutions can be enriching because it aligns with positive societal, organizational, and personal norms and values, but it can also be financially advantageous. The previously mentioned costs of implementing fairness metrics and auditing practices are smaller than the costs of addressing system limitations later in the development process. Implementing solutions at the design phase, compared to the testing phase or deployment stage, can ultimately cut costs by a significant amount (Dawson et al. 2010).

Considering another example, banks earn money by approving loans to reasonable credit risks, not by turning away performing loans. Biased algorithms that disadvantage a group of candidates and lock them out of the financial system, ultimately cap the revenue that banks could be accruing. This principle led the credit bureau Experian to create ‘Boost’ (Henry and Morris 2018)—a program that allows users with limited credit history to increase their scores with information about managing money. This allowed more than half of the users initially labelled as having a “poor” credit rating (simply due to lack of information), to be re-labelled as having a “fair” credit rating—giving them access to previously denied loans and allowing lenders to extend more credit to profitable customers.

DataRobot (2019)—in collaboration with the World Economic Forum—surveyed more than 350 U.S. and U.K.-based technology leaders to understand how organizations identify and mitigate bias in AI. Survey respondents included CIOs, IT directors, IT managers, data scientists and development leads involved in the design and deployment of AI systems. Crucially, 36% of respondents declared that their organizations suffered from AI bias. Damage was reported to be in the form of lost revenue by 62% of companies, lost customers in 61% of cases, lost employees due to AI bias in 43% of cases, and incurred legal fees due to a lawsuit or legal action relating to bias in 35% of cases (DataRobot 2019).

In addition to being “the right thing to do,” minimizing bias can therefore also be portrayed as an economic imperative—making organizations more profitable and productive (Wachter 2021) and enabling them to appear more alluring and trustworthy to customers/users who prefer to use products of companies that reflect their values (Zeno Group 2020).

### Laws and regulations

Another incentive to implement effective AI governance solutions is to improve business metrics such as regulatory preparedness. Laws and regulations can be powerful drivers of behavioral change. The increased discussion of the biased nature of AI systems has mobilized organizations and governments around the world to regulate how these technologies are developed and how they are utilized in decision-making contexts. Many existing and new regulatory frameworks create obligations to look for bias in AI due to their scope or by explicitly addressing the technology or concept. In the U.S, local, state, and federal regulations have already been enforced and members of Congress are introducing bills like the Algorithmic Accountability Act (2019) and the Algorithmic Fairness Act (2020), to promote ethical AI decision-making. While the federal laws do not explicitly target AI, the Federal Trade Commission (FTC) has, for example, issued a regulation noting that biased AI violates the Fair Credit Reporting Act (FCRA) given that the Equal Credit Opportunity Act (ECOA) renders it illegal for a company to utilize biased algorithms resulting in credit discrimination based on sensitive attributes such as national origin, race, or sex (Federal Trade Commission 2020, 2021).

In Europe, similar regulatory efforts can be observed. A recent regulation is the draft Artificial Intelligence Act or AI Act (2021). This is Europe’s first attempt to regulate AI comprehensively. Whilst this is still a draft, a clear regulatory trajectory can already be seen. The regulation classifies the different AI applications according to their risk, ranging from (i) low or minimal risk, (ii) limited risk, (iii) high risk, and (iv) unacceptable risk. The bulk of the regulation primarily aims at self-assessment procedures for operators of high-risk AI systems. Providers of high-risk systems (e.g., employment, critical infrastructure, education) need to undergo an ex-ante conformity assessment to test compliance with the regulation and the harmonized standards (Ebers et al. 2021). The draft requires that operators examine possible bias and ensure that the training, validation, and testing datasets are relevant, representative, free of errors, and complete (Art 10 (2) f and (3)), keep records (Art 12) and guarantee human oversight (Art 14). At this stage, it is still too early to describe the exact procedures and obligations the regulation will require of AI providers, but there will likely be several legal duties to test for and mitigate biases in high-risk systems.

There are, however, already frameworks in place that impact AI systems. In 2018 the General Data Protection Regulation (GDPR) came into force. Whilst this framework is primarily aimed at protecting privacy and regulating the use of data, some provisions have an impact on automated decision-making and AI. Art 22 of the GDPR stipulates that fully automated decisions that do have legal or similarly significant effects on data subjects warrant special legal safeguards. If such a decision is rendered, for example, a loan or employment decision, the data subject has a right to demand human intervention, express their point of view and contest the decision. In addition, Articles 13–15 GDPR grant individuals the right to obtain “meaningful information about the logic involved, as well as the significance and the envisaged consequences of such processing for the data subject” (Wachter et al. 2017, p.5). While these provisions do not directly address questions of bias, the heightened transparency requirements aim to increase individuals’ understanding of how and why automated decisions are made.

Moreover, the European Non-Discrimination Directives, even though not designed to regulate AI, are highly likely to have an increased impact on automated decision-making and the  deployment of AI. These directives prohibit direct and indirect discrimination. Direct discrimination prohibits the use of protected attributes such as gender, ethnicity, ability, or sexual orientation to make decisions in a protected sector, such as, for example, employment, or when offering goods and services (e.g., healthcare). Indirect discrimination is also prohibited, which refers to the use of an “apparently neutral provision, criterion, or practice [that] would put persons of a racial or ethnic origin at a particular disadvantage compared with other persons”. If such practices lead to unequal outcomes (e.g., unequal access to health services), the disparity would need to be legally justified (Hacker 2018; Zuiderveen Borgesius 2020).

The US has a similar system in place (Barocas and Selbst 2016). One of the crucial differences between these two systems is that the European system does not necessitate intent in direct discrimination cases. In other words, in the EU a person can be liable under law for direct (or indirect discrimination), even if they did not know about the discriminating nature of the system. This provision aims to encourage providers to investigate bias and discrimination as widely as possible, both before and after the deployment of an AI system,  as ignorance about biased performance or outcomes will not free them from liability (Wachter et al. 2020).

#### Psychology and legal compliance

Similarly to the aforementioned notions that making people aware of bias in technology is not enough to ensure people act to reduce this bias and creating technical solutions to mitigate bias is not enough to ensure people adopt these solutions—drawing up laws and regulations to regulate AI (although an important feat) does not in itself necessarily guarantee that people will comply with them, and it does not mean they will serve their intended purpose. Research within psychology and cognitive science has shown that compliance with laws is not only a cost–benefit calculation but is shaped by various factors such as social norms, social identity, imitation of others, and ethics (see Baier 2016; Bradford et al. 2015; Licht 2008; Nadler 2017)). Laws that reflect the affected community’s values and norms, enhance their ability to gain compliance (Acemoglu and Jackson 2017). As such, the processes mentioned so far, relating to values and norms, also play a role in moderating compliance with laws and legislation. This is a bi-directional relationship, with values and attitudes towards a certain behavior influencing how laws regulating that behavior are perceived—and laws being able to alter people’s attitudes towards the regulated behavior.

Research has found that laws can influence people's behavior through various means (see Bilz and Nadler 2014 for an overview), such as by leveraging their motivation to maintain the esteem of others. The most promising domains for changing behavior seem to be those in which people can be prompted to take the path of least resistance (Shenhav et al. 2017). This is in line with theories of diffusion of technology and innovation, which state (and find empirical support towards) that ‘ease of use’ and ‘workflow integration’ are crucial factors that predict attitudes towards technological change (Vale Martins et al. 2021; Zhou et al. 2019). Overall, what psychological research on legal compliance suggests is that even though conventional law and economics can explain differences in people’s willingness to engage in regulated behavior up to a certain point, people’s attitudes and beliefs about the behavior can also be altered (Bilz and Nadler 2014; Cialdini 2007; Roy 2021). If legal regulation can transmute the social meaning of behavior, and can change people’s perceptions regarding the desirability of this behavior, perhaps deep-rooted and widespread behavior change can be achieved. These notions should be considered when devising regulations and solutions to mitigate bias in AI, to maximize their effectiveness and maximise their adoption.

## Conclusions and suggestions

There is no shortage of research identifying biases in healthcare AI products and developing solutions—typically supported by ethical principles—to address these. However, there is a shortage of research investigating how to best motivate the widespread adoption of these solutions and promote fairness and transparency values amongst key developers and stakeholders of the technology. In this paper, we outlined how we can draw on theories stemming from social and motivational psychology to increase engagement with de-biasing and fairness-enhancing practices within the AI (healthcare) industry to promote sustained behavioral change. In doing so, we framed investment in fair AI as a value-expressive behavior, building on previous work that sees certain behaviors give expression to certain personally held values.

Ensuring that AI systems are designed and used legally, ethically, and safely requires organizations to not only have the right values and tools in place but also be able to effectively communicate these to individuals involved in the development and distribution of the systems. In this paper, we outlined how we can draw from motivational, and more broadly social, psychology when considering how to design effective communications. Researchers should empirically investigate the use of autonomy-supportive communication strategies as means of facilitating long-term behavior change (going beyond top-down directives) within the AI healthcare industry, in line with transparency and fairness values. Motivating individuals to care about fairness by design can ultimately act as a catalyst for internal and societal change. However, for these motivational strategies to be maximally effective, we argue they should also consider and incorporate the forces that are working for, and against, the desired behavior change. As such, they need to consider the existing norms and habits of a given environment and any known inhibitors and incentives of behavior change. Values are not activated in a vacuum but within an organizational, and broader societal, ecosystem. Research on optimizing value-activation within the healthcare AI industry to increase uptake of fairness by design practices should also inform research on best practices for creating legal mandates and regulations, and maximizing compliance with these.

Ultimately, developing real-world translational research requires extensive investment, and without care and time, researchers can draw hasty conclusions about real-world effects that, over broader periods, do not facilitate actual change. This type of research requires a process spanning multiple stems. The field must begin from robust basic research that is internally valid and reproducible and develop this work into field experiments and studies that extend scientific conclusions to real-world settings. The process requires commitment from academics and practitioners collaborating to draw theory-rich but applicable tests of research questions and hypotheses. It also requires academics from different disciplines to work together to produce broader research that considers context alongside individual differences.

## Data Availability

Data sharing is not applicable to this article as no datasets were generated or analysed for the current article.

## Footnotes

1 Other principles are beneficence, non-maleficence, autonomy, and explicability (see Floridi and Cowls, 2019).

2 Value alignment in AI refers to the process of ensuring that the values and ethical principles of the AI system is aligned with those of society, to ensure the outcomes of the system align with widely accepted ethical and moral principles and do not create negative outcomes for the individuals utilizing the systems, or society more broadly.

3 Excellent overviews of biases in healthcare AI have been provided in recent years (Cho 2021; Gerke et al. 2020; Lysaght et al. 2019; Norori et al. 2021; Sargent 2021).

4 The ‘Gender Shades Study’ is a renowned public audit conducted by Buolamwini and Gebru (2018) which evaluated bias present in automated facial analysis algorithms and datasets concerning phenotypic subgroups. When reviewing three commercial gender classification systems, (Buolamwini and Gebru 2018) found that the datasets used by these systems were overwhelmingly composed of lighter-skinned subjects. More problematically, even when the system used new facial analysis balanced datasets, the authors found that the systems had significantly higher misclassification rates for darker-skinned faces (darker-skinned females in particular).
